# AI language model applications for early diagnosis of childhood epilepsy based on unstructured first‐visit patient narratives: A cohort study

**DOI:** 10.1002/epd2.70109

**Published:** 2025-10-03

**Authors:** Jitse Loyens, Geertruida Slinger, Nynke Doornebal, Kees P. J. Braun, Eric van Diessen, Willem M. Otte

**Affiliations:** ^1^ Faculty of Medicine Utrecht University Utrecht the Netherlands; ^2^ Department of Child Neurology UMC Utrecht Brain Center, University Medical Center Utrecht and Utrecht University Utrecht The Netherlands; ^3^ Department of Pediatrics Martini Hospital Groningen the Netherlands; ^4^ Department of Pediatrics Franciscus Hospital Rotterdam the Netherlands

**Keywords:** childhood epilepsy, early diagnosis, large language models, natural language processing

## Abstract

**Objective:**

Language serves as an indispensable source of information for diagnosing epilepsy, and its computational analysis is increasingly explored. This study assessed – and compared – the diagnostic value of different language model applications in extracting information. The aim is to identify language patterns that may contain useful clinical information that is not overtly considered by the clinician from first‐visit documentation to improve the early diagnosis of childhood epilepsy.

**Methods:**

We analyzed 1561 patient letters from the first two seizure clinics. The dataset was divided into training and test sets to evaluate performance and generalizability. We employed an established Naïve Bayes model as a natural language processing technique and a sentence‐embedding (large language) model based on the Bidirectional Encoder Representations from Transformers (BERT) architecture. Both models analyzed anamnesis texts as noted by the treating physician only. Within the training sets, we identified predictive features consisting of keywords indicative of ‘epilepsy’ or ‘no epilepsy.’ Model outputs were compared to the clinician's final diagnosis (gold standard) after a two‐year follow‐up period. We computed accuracy, sensitivity, and specificity for both models.

**Results:**

The Naïve Bayes model achieved an accuracy of 0.73 (95% CI: 0.68–0.78), with a sensitivity of 0.79 (95% CI: 0.74–0.85) and a specificity of 0.62 (95% CI: 0.52–0.72). The sentence‐embedding model demonstrated comparable performance with an accuracy of 0.74 (95% CI: 0.68–0.79), a sensitivity of 0.74 (95% CI: 0.68–0.80), and a specificity of 0.73 (95% CI: 0.61–0.84).

**Significance:**

Both models demonstrated relatively good performance in diagnosing childhood epilepsy solely based on the first‐visit patient anamnesis text. Notably, the more advanced sentence‐embedding model showed no improvement over the computationally simpler Naïve Bayes model. This suggests that modeling of anamnesis data does depend on word order for this particular classification task. Further refinement and exploration of language models and computational linguistic approaches are necessary to enhance diagnostic accuracy in clinical practice.

**Key points**

- There is a growing trend to apply AI language models to improve patient identification, risk stratification, and outcome prediction.
- AI language models can help to diagnose childhood epilepsy solely based on first‐visit narratives.
- Exploring various AI language models is essential to assess if more complex and less interpretable methods offer meaningful advantages.
- Prospective studies are required to overcome the inherent limitations of AI language models.

## 1 INTRODUCTION

Epilepsy significantly impacts psychosocial well‐being and can adversely affect health‐related quality of life.^1^, ^2^ This impact is particularly concerning in children, where recurrent seizures can interfere with normal brain development, potentially leading to cognitive and behavioral impairments.^3^, ^4^, ^5^ These serious consequences underscore the critical importance of obtaining an early and accurate diagnosis of epilepsy.

Diagnosing epilepsy presents significant challenges due to its polymorphic nature.^6^, ^7^, ^8^ Research has shown that nearly half of the patients assessed for initial seizures were already experiencing recurrent, undiagnosed seizures at the time of evaluation.^4^, ^9^ While diagnostic time is typically brief for clearly identifiable cases of epilepsy, it can extend beyond a year for complex or ambiguous presentations.^10^, ^11^ This diagnostic uncertainty can have serious consequences: diagnostic delays expose children to ongoing seizures that may impair cognitive development, while false‐positive diagnoses can lead to unnecessary administration of antiseizure medications with potential adverse effects.^12^, ^13^, ^14^

Language plays a fundamental role in epilepsy diagnosis, treatment evaluation, and patient care management. Clinicians rely heavily on patient history and narrative to distill relevant clinical information.^15^ This makes collected text a rich and versatile medium for gaining deep insight into the patient's condition as required for a comprehensive approach to epilepsy care. Despite advances in ancillary investigations, clinical information from patient anamnesis remains indispensable for diagnosing and monitoring epilepsy.^16^, ^17^, ^18^ However, this wealth of information is often stored in electronic health records in an unstructured manner, limiting its optimal utilization in clinical decision‐making.^19^

The emergence of natural language processing (NLP) offers a promising solution for systematically processing this unstructured textual data. NLP, a form of artificial intelligence, specializes in the computational analysis of spoken and written language to identify general patterns and trends and extract relevant information.^20^, ^21^, ^22^ This involves converting unstructured text into a structured format and applying computational algorithms to analyze these structured features, enabling the retrieval of desired information.

In epilepsy research, there is a growing trend toward NLP applications, including patient identification, risk stratification, and outcome prediction. In clinical settings, NLP can contribute significantly to the early detection and classification of medical conditions, thereby reducing time to diagnosis and treatment.^19^ Recent advances have led to improved NLP models with new generative properties, known as large language models (LLMs).^23^, ^24^, ^25^ The essence of these models is a transformer architecture with an attention layer, a neural network model that processes text by assigning varying degrees of importance to different words through self‐attention mechanisms, allowing both an efficient representation and retrieval of relevant information in (textual) data.^25^ The potential of these more advanced language models and their applicability for early diagnosis of epilepsy is increasingly explored.^26^, ^27^, ^28^ This study aims to assess—and compare—the diagnostic value of different NLP approaches using medical letters from first consultations to facilitate the early diagnosis of childhood epilepsy.

## 2 METHODS

### 2.1 Dataset

Our analysis encompassed 1561 medical patient letters, with 1250 originating from University Medical Center Utrecht (UMCU) and 311 from Martini Hospital Groningen (MZG). We retrospectively collected data from children (age < 18 years) referred to the First Seizure Clinic (FSC) between 2008 and May 2022. These data were originally collected for previously published studies focusing on prediction model development for childhood epilepsy and the clinical characteristics and diagnoses of children referred to an FSC.^7^, ^29^ The institutional ethics committee of both hospitals approved the use of anonymized retrospective data for research purposes without informed consent.

All patient letters were either written or supervised by experienced pediatric neurologists and were available in an electronic format. These medical letters typically contain all information available or collected during the FSC visit (i.e., anamnesis, ancillary investigations, conclusions, treatment plans, and clinical considerations). This medical letter is the documentation eventually sent to the referring physician. For each patient, we included both the initial diagnosis (established after the FSC consultation) and the final diagnosis (reached through consensus among doctors and after ancillary investigations – if needed – at the latest follow‐up, recorded within a two‐year period following presentation).

Follow‐up occurred for children with inconclusive diagnoses at the first consultation and for those initially diagnosed with epilepsy. Children whose epilepsy diagnosis was definitely ruled out at presentation were referred back to their referral specialist or general practitioner for follow‐up. Ancillary investigations used to establish final diagnoses included electroencephalography (EEG), neuroimaging, video monitoring, metabolic testing, and genetic analyses as clinically indicated. The specific testing performed varied based on individual clinical presentation and followed standard diagnostic protocols for pediatric epilepsy evaluation.

**FIGURE 1:**
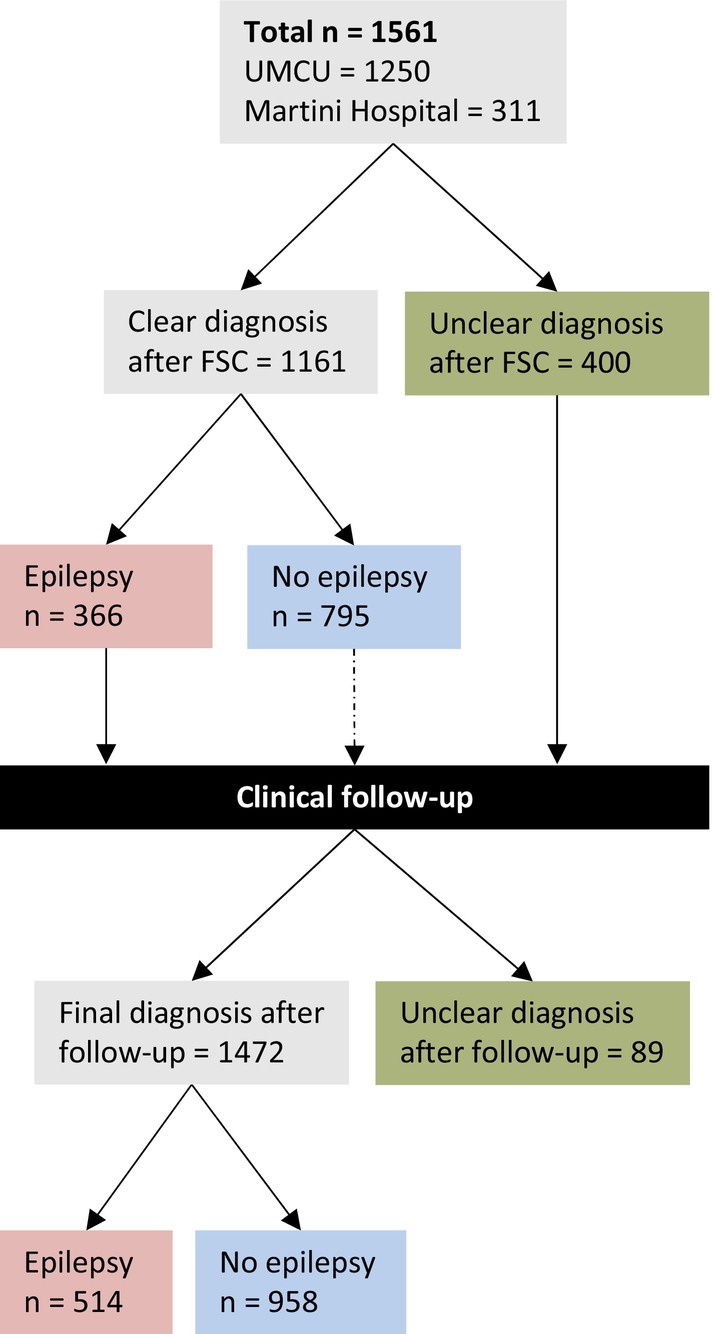
Flowchart illustrating the diagnostic pathway for children referred to the FSC. The flowchart outlines the process from the first FSC consultation to the final diagnosis, including follow‐up procedures. The diagnoses are categorized as ‘epilepsy’, ‘no epilepsy’, or ‘unclear’.

Both initial and final diagnoses were categorized into three groups: ‘epilepsy’, ‘no epilepsy’, and ‘unclear’ (Figure 1) and served as the model's outcome. All epilepsy diagnoses were established according to the International League Against Epilepsy definition of epilepsy.^30^ A diagnosis was classified as ‘unclear’ at the initial stage if ancillary investigations were deemed necessary to confirm or reject the epilepsy diagnosis. The final diagnosis was classified as ‘unclear’ if, despite further investigations, uncertainty remained about whether the events were indeed epilepsy‐related.^7^

### 2.2 Study design

We conducted a retrospective analysis of the letters to assess the clinical value of language models for early diagnosis of childhood epilepsy. This was achieved through binary text classification, specifically by training classification models based on textual features and predicting the class of new texts within the ‘epilepsy’ and ‘no epilepsy’ patients. To reduce interpretative bias, we exclusively used textual information from the patient's history (anamnesis – the clinical history as reported by the patient or caregiver), excluding information from ancillary investigations, conclusions, treatment plans, and clinical considerations. All patient data were anonymized prior to analysis by removing all personal identifiers, including names, addresses, dates of birth, and unique identifying numbers. This process was conducted by medical professionals familiar with the data to ensure the clinical context was preserved. The Dutch texts were analyzed in their original language without translation, as our NLP approaches can process Dutch language input. This approach avoided potential information loss or distortion that might occur during translation.

Our study involved two distinct analyses. In analysis A, we combined data from both hospitals and randomly divided it into a training set (80%; 1173 subjects) and a test set (20%; 293 subjects). To ensure representative distribution of final diagnoses in both sets, we applied stratification based on the final diagnosis groups. In analysis B, we created a separate test set comprising all 316 subjects that were classified as unclear after initial FSC evaluation but assigned a definitive diagnosis after clinical follow‐up. This second analysis aimed to determine whether the model could accurately classify initially unclear – and clinically more complicated – cases as either having epilepsy or not (Figure 1). Thus, the models were trained to predict the final diagnosis (after follow‐up) rather than the initial diagnosis. We chose this approach because the final diagnosis represents the most accurate clinical assessment after sufficient observation and testing. The initial diagnoses (particularly those classified as ‘unclear’) were not used as training labels but rather to stratify our analyses into groups A and B to evaluate model performance on different clinical scenarios.

Stratification by final diagnosis group was implemented by performing a stratified random split (caret package R), ensuring that the proportions of epilepsy, no epilepsy, and unclear cases in both training and test sets matched those in the overall dataset. This approach maintained the natural class distribution across all data partitions while ensuring that less common diagnosis groups were adequately represented in both training and test sets. To account for potential differences between the two hospital datasets (UMCU and MZG), we ensured balanced representation of data from both centers in the training and test sets through stratified sampling based on both hospital source and diagnosis. While formal stratification by site (such as through random effects models) was not implemented due to the non‐parametric nature of our models, our sampling approach ensured that both hospitals' documentation styles were represented proportionally in both training and testing data.

The letter corpus exhibited considerable variation in textual length, ranging from 63 to 1070 words, with a median of 400 words and a mean of 414 words. Six cases (three from the UMC Utrecht; four female subjects) were excluded from the training set due to their succinct nature, consisting of only single sentences in their amnestic report. For the readability of the study, we translated all words into English. A list of the most relevant original items (words or word combinations) is provided (Table S1). A TRIPOD AI checklist was added as guidance (Data S1).

### 2.3 Naïve Bayes model

**FIGURE 2:**
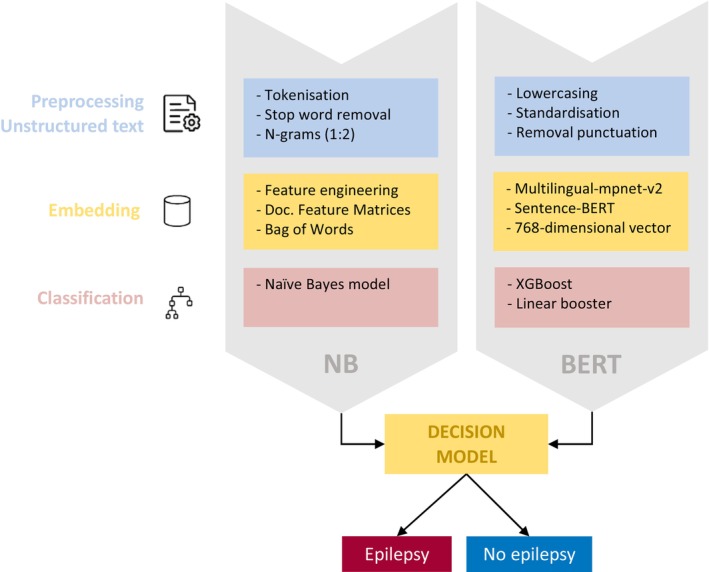
Workflow for classifying ‘epilepsy’ or ‘no epilepsy’ diagnosis based on unstructured letters from the first consultations for both models: NB = Naïve Bayes and BERT. The process consists of three main stages: preprocessing, embedding, and classification model.

We used a Naïve Bayes classifier as an NLP approach, given its simplicity and effectiveness in text classification. The essence of the model is the application of Bayes' theorem, assuming a strong independence between features.^31^, ^32^ Model development contained three phases: data preprocessing, data analysis with feature selection, and classification (Figure 2).

#### 2.3.1 Data preprocessing

Preprocessing encompasses several key steps, including corpus creation, tokenization, data cleaning, lowercasing, n‐gram generation, and stop word removal. Creating a corpus involves collecting and organizing a substantial amount of textual data in a structured manner to facilitate systematic analysis and processing. Text was then divided into tokens (i.e., words) through tokenization.^33^ Undesired characters, such as punctuation marks, symbols, URLs, and separators, were omitted (data cleaning). Lowercasing converted all characters in the text to lowercase letters, ensuring consistency across the tokens. Afterwards, n‐grams were generated, with a maximum n‐value of 2. N‐grams are sequences of consecutive words and will be used as features for the text classification model.^34^ It was decided to generate unigrams (single words such as “jerks”) and bigrams (pairs of consecutive words such as “no_jerks”). The final step involved removing stop words from the generated n‐grams. Removing stop words after generating n‐grams ensures that some meaningful bigrams are retained, even if they contain stop words (e.g., “no_fever” may be retained while “no” and “fever” may individually be stop words). Stop words contain common words, including prepositions, personal pronouns, units, and auxiliary verbs that lack informativeness and may interfere with model development. We selectively retained pronouns in bigrams when they formed part of clinically relevant phrases. For example, while “her” would typically be removed as a standalone word, bigrams like “her_mouth” were preserved due to their potential clinical significance in describing observed symptoms. This selective approach allowed us to retain contextually important information while still removing uninformative stop words. After the preprocessing step, the dataset was split into training and test sets.

#### 2.3.2 Data analysis

We created a document‐feature matrix (DFM) for the training set to enable structured analysis of text data, representing documents (letters) as rows and features (i.e., all n‐grams) as columns. The matrix values represented the frequency of a feature in each letter, creating a Bag‐of‐Words (BoW) model.^35^ In this model, the input text is represented as a collection of words, disregarding the order in which they appear. We applied Term Frequency‐Inverse Document Frequency (TF‐IDF) to weigh features based on their frequency in individual letters. TF‐IDF reduces the influence of frequently occurring features while emphasizing more informative ones.^36^

#### 2.3.3 Feature selection

Feature selection was achieved through Recursive Feature Elimination (RFE) with 5‐fold cross‐validation. RFE identified the top 300 features that were most informative for the model's performance. A selection of 300 features was based on theoretical and practical reasons. Firstly, we wanted to follow the rule of thumb that recommends one feature per ten cases to minimize overfitting and optimize model performance. As the dataset is of medium size, we adjusted this rule to one feature per five cases, resulting in the selection of 300 features. Secondly, the literature supports this selection, as studies frequently use between 200 and 300 features to capture significant patterns while minimizing noise, thereby enhancing the robustness and generalizability of the model.^37^, ^38^, ^39^ Thirdly, fewer features improve computational efficiency, making the model more practical for implementation. Moreover, fewer features improve the model's interpretability and transparency, facilitating a better understanding of which variables contribute to its predictions. As a hyperparameter for the Naïve Bayes model, the smoothing parameter (α) was added to prevent zero probabilities. Importantly, all feature engineering steps – the creation of DFM, application of TF‐IDF transformations, and feature selection through RFE – were performed exclusively on the training set to prevent data leakage. The test set was only used for final model evaluation after all model parameters and features were determined.

### 2.4 Sentence‐embedding model and subsequent classification

This study also employed a classification model to predict epilepsy diagnoses based on patient text records that takes—in contrast to the BoW approach—word sequence into account. First, textual data underwent systematic preprocessing. Initial preprocessing steps included case normalization to lowercase, standardization of special characters to their lexical equivalents, removal of extraneous punctuation marks, and normalization of whitespaces. Next, the processed texts were then embedded using a freely available multilingual embedding (i.e., the paraphrase‐multilingual‐mpnet‐base‐v2 transformer model; https://huggingface.co/sentence‐transformers/paraphrase‐multilingual‐mpnet‐base‐v2). The embedding model implements the Sentence‐BERT architecture to generate contextualized 768‐dimensional semantic vector representations for each text, irrespective of its length.^40^ This embedding model was selected for its capacity to preserve both sequential word order information and cross‐lingual semantic relationships. Third, the resulting high‐dimensional embeddings served as input features for a gradient boosting classifier implemented through the XGBoost framework in R.^41^ The binary classification model employed a linear booster with default hyperparameters, leveraging sequential tree building to iteratively optimize the prediction objective while maintaining computational efficiency.

### 2.5 Performance evaluation

Performance evaluation utilized confusion matrices to compare actual and predicted classifications through decision statistics from contingency tables. We compared each model's output to the clinician's final diagnosis (gold standard). Key performance metrics included: accuracy (i.e., the proportion of correct classifications), sensitivity (true positive rate), and specificity (true negative rate). Sensitivity and positive predictive value specifically refer to correctly identifying epilepsy cases, while specificity and negative predictive value refer to correctly identifying ‘no epilepsy’ cases. Clinical consequences vary by error type: threshold‐independent performance measures (such as Area Under the Curve) treat false positives and false negatives equally, but in epilepsy diagnosis, these errors have different clinical implications.^4^, ^12^, ^14^ Missing epilepsy (false negative) may delay critical treatment and expose children to ongoing seizures, while overdiagnosis (false positive) may lead to unnecessary medication with potential adverse effects.^42^ Our threshold‐specific metrics allow evaluation of these distinct clinical scenarios. McNemar's test was performed to statistically compare the performance of both models. All evaluation analyses were performed using R software, version 4.4.0.

## 3 RESULTS

### 3.1 Data characteristics

The median age at the first seizure was 4.5 years (95% CI: 4.0–4.9, range 1 m – 17.8 years). The majority of patients were male (54.6%). After the first consultation, 366 (23.4%) diagnoses were classified as ‘epilepsy’, 795 (50.9%) as ‘no epilepsy’, and 400 (25.6%) as ‘unclear’. According to the final diagnoses, 514 (32.9%) diagnoses were classified as ‘epilepsy’ (413 from UMCU and 101 from MZG), 958 (61.4%) as ‘no epilepsy’ (767 from UMCU and 191 from MZG), and 89 (5.7%) as ‘unclear’ (70 from UMCU and 19 from MZG). The data characteristics are presented (Table S2). Specific information on the exact diagnosis can be found elsewhere.^7^

### 3.2 Most important features

**FIGURE 3:**
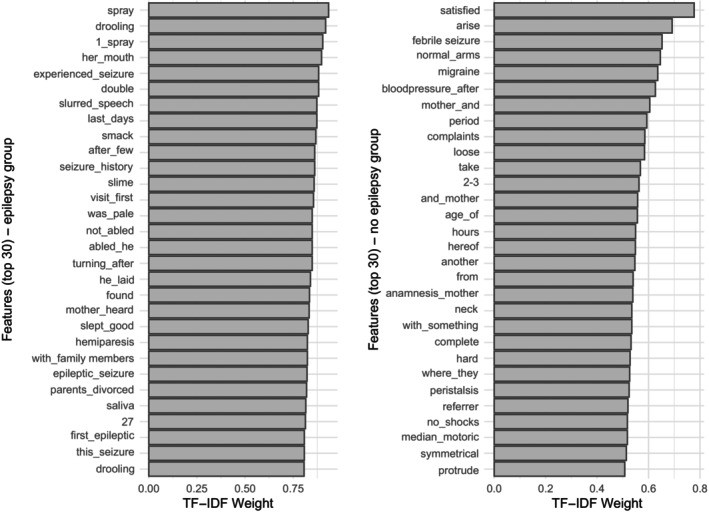
A graphical representation of the most prevalent features for each condition. TF‐IDF, Term Frequency‐Inverse Document Frequency; to weigh features based on their frequency in individual letters.

The *Term Frequency‐Inverse Document Frequency* identified several key features most characteristic of the epilepsy classification texts. Notable predictive n‐gram features included:

“spray”, “drooled”, “her_mouth”, “experienced_seizure”, “slurred_speech”, and “last_days”. Some features directly reflected clinical observations or descriptions that frequently appear in letters from epilepsy patients, while others, such as “last_days” or “her_mouth”, have a less obvious connection to epilepsy. Lists of the most important features for epilepsy, as well as the control group, are provided in Figure 3.

### 3.3 Classification model performance

#### 3.3.1 Analysis a (all patients, with a final diagnosis only)

The performance of the language models was evaluated on a test set of 293 letters. The Naïve Bayes model correctly identified 62 (21.2%) letters as positive and 153 (52.2%) as negative, resulting in 40 (13.7%) false positives and 30 (10.2%) false negatives, with an overall accuracy of 0.73 (95% CI: 0.68–0.78). The Sentence‐embedding model correctly identified 40 (13.7%) letters as positive and 176 (60.1%) as negative, classifying 62 (21.2%) false positives and 15 (5.1%) false negatives, with an overall accuracy of 0.74 (95% CI: 0.68–0.79). An overview including sensitivity, specificity, positive predictive value (PPV), and negative predictive value (NPV) is provided (Table 1).

**TABLE 1:** Model performance metrics for epilepsy diagnosis: Test sets. Analysis A (all patients, with a final diagnosis only). Analysis B (patients with FSC diagnosis unclear only). NB = Naïve Bayes. Between parentheses = 95% Confidence Interval.

Analysis	Model	Accuracy	Sensitivity	Specificity	PPV	NPV
A	NB	0.73 (0.68–0.78)	0.79 (0.74–0.85)	0.62 (0.52–0.72)	0.80 (0.74–0.86)	0.61 (0.51–0.70)
	BERT	0.74 (0.68–0.79)	0.74 (0.68–0.80)	0.73 (0.61–0.84)	0.92 (0.88–0.96)	0.39 (0.30–0.49)
B	NB	0.74 (0.69–0.79)	0.82 (0.77–0.88)	0.57 (0.47–0.66)	0.79 (0.74–0.84)	0.62 (0.52–0.72)
	BERT	0.72 (0.67–0.77)	0.75 (0.70–0.80)	0.57 (0.44–0.71)	0.89 (0.85–0.94)	0.32 (0.23–0.41)

#### 3.3.2 Analysis B (patients with FSC diagnosis unclear only)

The performance of the language models was evaluated on a test set of 316 letters with an ‘unclear’ diagnosis at the time of FSC analysis. The Naïve Bayes model correctly identified 60 (19.0%) letters as positive and 173 (54.7%) as negative, resulting in 37 (11.7%) false positives and 46 (14.6%) false negatives, with an overall accuracy of 0.74 (95% CI: 0.69–0.79). The Sentence‐embedding model correctly identified 31 (10.0%) letters as positive and 196 (62.0%) as negative, classifying 66 (20.9%) false positives and 23 (7.3%) false negatives, with an overall accuracy of 0.72 (95% CI: 0.67–0.77). An overview including sensitivity, specificity, PPV, and NPV is provided (Table 1). It is noteworthy that Analysis B, which focused on the more challenging cases that remained diagnostically uncertain after initial clinical evaluation, still achieved performance metrics comparable to those in Analysis A. This suggests robust model generalization even to clinically ambiguous presentations. We performed McNemar's test to statistically compare the performance of both models. No statistical difference was found between the Naïve Bayes model and the sentence‐embedding model in both Analysis A and B (*χ* ^2^ = 0.06, *df* = 1, *p*‐value = 0.804 and *χ* ^2^ = 0.55, *df* = 1, *p*‐value = 0.461).

## 4 DISCUSSION

This study evaluated and compared two different language model applications for the early diagnosis of childhood epilepsy through automated analysis of first‐visit documentation. Our findings revealed comparable performance between the simpler Naïve Bayes model and the more advanced sentence‐embedding model, with both achieving moderate to good diagnostic accuracy. Notably, both models demonstrated higher sensitivity than specificity across all analyses. Translating our findings to clinical practice, the sentence‐embedding model's high PPV (0.92) indicates that when this model predicts epilepsy, it is correct approximately 92% of the time. This could significantly impact clinical decision‐making by providing greater confidence in positive epilepsy predictions, potentially expediting treatment decisions. Conversely, the model's lower NPV (0.39) means that negative predictions should be interpreted cautiously, as approximately 61% of patients predicted not to have epilepsy may actually have the condition. These scores are similar for both analyses A and B, with a higher NPV value for the Naïve Bayes approach. This suggests that while the model may efficiently identify likely epilepsy cases, it should not be used in isolation to rule out epilepsy. These predictive values align with the ILAE definition threshold of 60% likelihood for epilepsy diagnosis, suggesting potential clinical utility when appropriately integrated with existing diagnostic workflows.^43^ The comparable performance achieved in Analysis B is particularly promising, as these cases represent the diagnostically challenging subset where computational support could provide the most clinical value. Previous research has established the value of NLP in various aspects of epilepsy care, including patient identification,^44^, ^45^, ^46^ information retrieval,^47^, ^48^, ^49^ and coping strategies.^50^, ^51^ Recent studies have begun exploring language applications in early clinical phenotyping and genetic epilepsies.^52^, ^53^ However, our study uniquely addressed the specific challenges of early childhood epilepsy diagnosis, where textual analysis holds particular promise given the heterogeneous presentation of symptoms.

The performance metrics should be considered in the context of the model performance: our models relied solely on patient narratives, deliberately excluding information from EEG reports, clinical evaluations, and medical conclusions. From this perspective, our findings suggest that language models might have potential as supportive tools in the early phase of clinical evaluation for children with suspected epilepsy, though significant validation and integration work would be needed before any clinical implementation. These approaches should be viewed as potential complementary tools to enhance, rather than replace, expert clinical assessment. Interestingly, the transformer‐based Sentence‐embedding model – which takes word order into account – demonstrated an improved PPV but lower NPV in respect to the Naïve Bayes model. The Naïve Bayes model is regarded as a robust classification model, even when working with limited data and feature sets.^31^ Transformer‐based language models perhaps require longer text sequences to effectively recognize desired patterns, particularly in cases with less variation in language utilization. With limited text input, simpler models can offer greater practical value in practice, where speed, simplicity, and apprehensibility often take precedence in the implementation within clinical workflows. In clinical implementation contexts, model interpretability, computational efficiency, and seamless integration with existing workflows are important considerations that may favor simpler approaches, provided they offer comparable performance.^54^, ^55^

Interestingly, our study revealed that both obvious and less obvious words (or combinations) are of additional value for correct classification. The use of epilepsy‐related terminology could reflect the physician's (implicit) evaluation of the clinical case during consultation. Unrelated word combinations with no obvious relation to epilepsy that were classified as relevant features for model development may represent either underlying linguistic patterns common in epilepsy‐related letters or potential limitations in the model's feature selection process. Furthermore, we did not implement synonym mapping for medical terms (e.g., “drooled” and “drooling”), which may have led to unnecessary feature dimensionality. The presence of pronouns and gender‐specific terms among predictive features suggests that details about who provided the history (e.g., “anamnesis_mother”) may carry predictive value, perhaps reflecting differences in observational detail or reporting based on the historian's relationship to the patient. Previous efforts in the field have revealed similar insights into the non‐semantic evaluation of patient history and showed that hesitations and formulation efforts might be of additional value when diagnosing epilepsy.^56^, ^57^ Future research efforts should therefore include a comparison of different language model approaches to further elucidate the true value of this implicit language information for diagnosing epilepsy.

This study benefits from a substantial and diverse dataset collected from two hospitals, enhancing the robustness and generalizability of the model's results. The unequal sample sizes between our two hospital datasets limited our ability to perform robust between‐hospital validation. Future work with more balanced multi‐center data should evaluate cross‐institutional generalizability more thoroughly. The retrospective nature and moderate size of our dataset are, however, potential contributors to the limited specificity and NPV of both models, thereby increasing the chance of incorrectly identifying children as having epilepsy cases and false negatives, respectively. Furthermore, performance was significantly higher on the training sets (not reported) compared to the test sets, indicating potential overfitting. Overfitting occurs when the model learns the textual details and noise in the training data, which impairs its generalizability to new data. This can result from excessive noise, an excessive number of features, irrelevant features, or insufficient training data. Equally important to consider is the imbalanced dataset we used (uneven class distribution), predominantly consisting of letters from children with a ‘no epilepsy’ diagnosis. Imbalanced data can hinder a model's ability to learn the minority class, as (language) models often exhibit a preference for the majority class.^58^ As stated earlier, we deliberately restricted our analysis to the anamnesis text alone, excluding information from ancillary investigations, to specifically evaluate the predictive value of narrative clinical history in isolation. This approach allowed us to assess whether language patterns in patient histories contain sufficient signal for early diagnostic support, potentially enabling earlier screening before comprehensive testing is complete. We acknowledge that a complete clinical assessment would incorporate additional data sources, but will require a whole different study design.

From a model perspective, few limitations should be mentioned. A Naïve Bayes model is relatively limited in its capacity to learn complex (textual) relations.^31^ The model does not adequately account for word order or combinations of words, potentially resulting in misinterpretations of negations and the overall meaning within clinical text. Confounding factors like typographical errors, abbreviations, double negations, and letters written by multiple authors can adversely affect the classification process. We selected a maximum n‐gram value of 2 based on preliminary analyses and data size considerations. While longer n‐grams can capture more complex linguistic patterns, particularly in languages like Dutch, where negation may appear at different sentence positions, they require substantially larger datasets to avoid sparsity issues. Our dataset size of approximately 1500 documents was insufficient to effectively model higher‐order n‐grams without overfitting. Future work with larger datasets could explore the value of longer n‐gram sequences. Additionally, RFE was applied to a subset of the top 8000 features (i.e., 300) due to computational constraints, possibly excluding relevant features. A general limitation of feature selection is the possible omission of rare but significant features, particularly in the context of rare diseases or syndromes. Technically, more word‐order‐oriented models could (partially) overcome the aforementioned model limitations due to their transformer architecture in which meaningful textual relations are represented internally. Mechanisms that drive these models to achieve such model properties remain difficult to grasp, prohibiting a better understanding of these models.^59^, ^60^ A final limitation of our study is the lack of comparison with contemporary large language models (LLMs). While BERT‐based sentence embeddings represent a significant advance over bag‐of‐words approaches, they do not reflect the latest developments in language model architecture. Forthcoming work should evaluate how Generative Pre‐trained Transformer (GPT)‐like models compare to our approaches, particularly in identifying subtle linguistic patterns in clinical narratives. Our current comparison still provides valuable insights into the role of word order in this specific classification task.

Future research should incorporate a prospective design to explore the clinical applicability. Prospective studies enhance variable control, minimize data noise, and allow real‐time language capture, thereby reducing biases and missing data. Importantly, it will reduce the presence of epilepsy‐related terminology that reflects a physician's implicit case evaluation, potentially leading to an informational bias. A prospective design would also allow capturing a recorded – instead of a written – patient history that would inevitably lead to new potential hidden language domain sources (e.g., phonology, prosody, syntax use) to improve epilepsy diagnosis.^56^, ^57^ This could be particularly beneficial for LLMs as these models excel in retrieving ‘hidden’ textual associations that might be used for classification. Enhancing algorithms, refining feature selection, and utilizing larger, more diverse datasets are essential to improve diagnostic accuracy. Apart from methodological improvements, integration of language‐based classification models with existing clinical diagnostic tools in epilepsy care would be the next step to explore its actual clinical value.^29^, ^61^

## 5 CONCLUSIONS

Language models have the potential to achieve meaningful performance in supporting early childhood epilepsy diagnosis, even when limited to first‐visit documentation. The comparable performance between Naïve Bayes and more sophisticated transformer‐based language models suggests that simpler, more interpretable models may be preferable for initial clinical applications as long as the input data is limited in size and complexity. While further refinement is needed, these findings support the potential value of computational linguistic approaches in improving early epilepsy diagnosis and patient care. The higher sensitivity and PPV suggest that both models are particularly useful for correctly identifying epilepsy cases. As these methods continue to evolve, their integration into clinical practice could provide valuable decision support for clinicians while maintaining the essential role of clinical expertise in final diagnostic decisions.

## AUTHOR CONTRIBUTIONS

E.v.D. and W.M.O. initiated the study. J.L., G.S., N.D., and E.v.D. collected the data. All authors contributed to the analysis and interpretation of the data and contributed to drafting and finalizing the manuscript.

## CONFLICT OF INTEREST STATEMENT

None of the authors has any conflicts of interest to disclose. We confirm that we have read the journal's position on issues involved in ethical publication and that this report is consistent with those guidelines.

**Test yourself**

1. Which of the following statement is true on early and accurate diagnosis of childhood epilepsy in clinical practice?
  - Most children do not outgrow seizures without treatment
  - Early diagnosis prevents the need for genetic testing
  - Ongoing seizures may impair brain development and cognition
  - Childhood epilepsy can be reliably excluded or diagnosed with a first EEG
2. What is the primary advantage of using AI language models in the diagnosis of epilepsy according to the article?
  - To eliminate the need for EEG testing
  - To capture overlooked clinical information from unstructured patient history
  - To effectively diagnose in diagnosing childhood onset epilepsy
  - To replace the need for a clinical neurologist
3. A 6‐year‐old child presents with ambiguous semiology following an episode of unresponsiveness. Based on the study findings, which statement best describes how AI language models can support clinical decision‐making at this stage?
  - The model can replace EEG testing in this case
  - AI language models can help flag likely epilepsy cases early, guiding further testing
  - A negative result from the model should be taken as definitive
  - AI language models are unsuitable for uncertain clinical presentations
*Answers may be found in the* *supporting information*

## Supporting information


Table S1.

Data S1.

Appendix S1.

## Data Availability

The data that support the findings of this study are available from the corresponding author upon reasonable request.
