# Supplementary material for: AI language model applications for early diagnosis of childhood epilepsy based on unstructured first‐visit patient narratives: A cohort study
Source: Epileptic Disord. 2025 Oct 3;27(6):1263–74. doi: 10.1002/epd2.70109 (PMC12747707; doi:10.1002/epd2.70109)
Supplement: Supplementary file 1 — Table S1. [file EPD2-27-1263-s001.docx]

# Appendix

# Table S1. A complete translation list of most prevalent features (Dutch-English)

| **Features epilepsy group** | | **Features no epilepsy group** | |
| --- | --- | --- | --- |
| ***Dutch*** | ***English*** | ***Dutch*** | ***English*** |
| Spray | Spray | Tevreden | Satisfied |
| Kwijlde | Drooled | Ontstaan | Arise |
| 1_spray | 1_spray | Koortsstuip | Febrile seizure |
| Haar_mond | Her_mouth | Normaal_armen | Normal_arms |
| Insult_doorgemaakt | Experienced_seizure | Migraine | Migraine |
| Dubbele | Double | RR_na | Bloodpressure_after |
| Dubbele_tong | Slurred_speech | Moeder_en | Mother_and |
| Afgelopen_dagen | Last_days | Periode | Period |
| Smakt | Smack | Klachten | Complaints |
| Na_paar | After_few | Los | Loose |
| Insult_voorgeschiedenis | Seizure_history | Pakken | Take |
| Slijm | Slime | 2-3 | 2-3 |
| Komst_eerst | Visit_first | En_moeder | And_mother |
| Was_bleek | Was_pale | Leeftijd_van | Age_of |
| Niet_kon | Not_abled | Uren | Hours |
| Kon_hij | Abled_he | Hierover | Hereof |
| Draaien_naar | Turning_after | Een_andere | Another |
| Hij_lag | He_laid | Vanaf | From |
| Trof | Found | Anamnese_moeder | Anamnesis_mother |
| Moeder_hoorde | Mother_heard | Nek | Neck |
| Goed_geslapen | Slept_good | Met_wat | With_something |
| Hemiparese | Hemiparesis | Geheel | Complete |
| Bij_familieleden | With_family members | Hard | Hard |
| Epileptisch_insult | Epileptic_seizure | Waarbij_ze | Where_they |
| Ouders_gescheiden | Parents_divorced | Peristaltiek | Peristalsis |
| Speeksel | Saliva | Verwijzer | Refferer |
| 27 | 27 | Geen_schokken | No_shocks |
| Eerste_epileptische | First_epileptic | Mediaan_motoriek | Median_motoric |
| Deze_aanval | This_seizure | Symmetrisch | Symmetrical |
| Kwijlen | Drooling | Uitsteken | Protrude |

**Table S2.** Baseline characteristics of the data.

| **Characteristics** | **Total, N (%)** |
| --- | --- |
| ***Medical letters after the first consultation*** |  |
| UMCU | 1250 (80.1) |
| MZG | 311 (19.9) |
| Sex |  |
| Female | 708 (45.4) |
| Male | 853 (54.6) |
| Age |  |
| Median | 4,5 |
| Mean | 5,9 |
| Highest age | 17.8 |
| Lowest age | 0 |
| ***Epilepsy diagnosis after first consultation*** |  |
| Epilepsy | 366 (23.5) |
| No epilepsy | 795 (50.9) |
| Unclear | 400 (25.6) |
| ***Epilepsy diagnosis after two years of follow-up*** |  |
| Epilepsy | 514 (32.9) |
| No epilepsy | 958 (61.4) |
| Unclear | 89 (5.7) |
| ***Epilepsy diagnosis after two years of follow-up from UMCU*** |  |
| Epilepsy | 413 (33.0) |
| No epilepsy | 767 (61.4) |
| Unclear | 70 (5.6) |
| ***Epilepsy diagnosis after two years of follow-up from MZG*** |  |
| Epilepsy | 101 (32.5) |
| No epilepsy | 191 (61.4) |
| Unclear | 19 (6.1) |

Abbreviations: N = total number, UMCU = University Medical Center Utrecht, MZG = Martini Hospital Groningen.
