# Supplementary material for: AI language model applications for early diagnosis of childhood epilepsy based on unstructured first‐visit patient narratives: A cohort study
Source: Epileptic Disord. 2025 Oct 3;27(6):1263–74. doi: 10.1002/epd2.70109 (PMC12747707; doi:10.1002/epd2.70109)
Supplement: Supplementary file 3 — Appendix S1. [file EPD2-27-1263-s003.docx]

**TEST YOURSELF**

**Answers:**

**1. C**

**2. B**

**3. C**
